# Nationwide Results of COVID-19 Contact Tracing in South Korea: Individual Participant Data From an Epidemiological Survey

**DOI:** 10.2196/20992

**Published:** 2020-08-25

**Authors:** Seung Won Lee, Woon Tak Yuh, Jee Myung Yang, Yoon-Sik Cho, In Kyung Yoo, Hyun Yong Koh, Dominic Marshall, Donghwan Oh, Eun Kyo Ha, Man Yong Han, Dong Keon Yon

**Affiliations:** 1 Department of Data Science Sejong University College of Software Convergence Seoul Republic of Korea; 2 Department of Neurosurgery Seoul National University Hospital Seoul Republic of Korea; 3 Department of Ophthalmology Asan Medical Center Seoul Republic of Korea; 4 Department of Gastroenterology CHA Bundang Medical Center Seongnam Republic of Korea; 5 FM Kirby Neurobiology Center Boston Children's Hospital Harvard Medical School Boston, MA United States; 6 Critical Care Research Group Nuffield Department of Clinical Neurosciences Oxford United Kingdom; 7 Department of Internal Medicine Gangnam Severance Hospital Yonsei University College of Medicine Seoul Republic of Korea; 8 Department of Pediatrics Kangnam Sacred Heart Hospital Hallym University College of Medicine Seoul Republic of Korea; 9 Department of Pediatrics CHA Bundang Medical Center CHA University School of Medicine Seongnam Republic of Korea; 10 Armed Force Medical Command Republic of Korea Armed Forces Seongnam Republic of Korea

**Keywords:** COVID-19, contact tracing, coronavirus, South Korea, survey, health data, epidemiology, transmission

## Abstract

**Background:**

Evidence regarding the effectiveness of contact tracing of COVID-19 and the related social distancing is limited and inconclusive.

**Objective:**

This study aims to investigate the epidemiological characteristics of SARS-CoV-2 transmission in South Korea and evaluate whether a social distancing campaign is effective in mitigating the spread of COVID-19.

**Methods:**

We used contract tracing data to investigate the epidemic characteristics of SARS-CoV-2 transmission in South Korea and evaluate whether a social distancing campaign was effective in mitigating the spread of COVID-19. We calculated the mortality rate for COVID-19 by infection type (cluster vs noncluster) and tested whether new confirmed COVID-19 trends changed after a social distancing campaign.

**Results:**

There were 2537 patients with confirmed COVID-19 who completed the epidemiologic survey: 1305 (51.4%) cluster cases and 1232 (48.6%) noncluster cases. The mortality rate was significantly higher in cluster cases linked to medical facilities (11/143, 7.70% vs 5/1232, 0.41%; adjusted percentage difference 7.99%; 95% CI 5.83 to 10.14) and long-term care facilities (19/221, 8.60% vs 5/1232, 0.41%; adjusted percentage difference 7.56%; 95% CI 5.66 to 9.47) than in noncluster cases. The change in trends of newly confirmed COVID-19 cases before and after the social distancing campaign was significantly negative in the entire cohort (adjusted trend difference –2.28; 95% CI –3.88 to –0.68) and the cluster infection group (adjusted trend difference –0.96; 95% CI –1.83 to –0.09).

**Conclusions:**

In a nationwide contact tracing study in South Korea, COVID-19 linked to medical and long-term care facilities significantly increased the risk of mortality compared to noncluster COVID-19. A social distancing campaign decreased the spread of COVID-19 in South Korea and differentially affected cluster infections of SARS-CoV-2.

## Introduction

The novel coronavirus that emerged in Wuhan, China, termed SARS-CoV-2, has caused a rapidly spreading outbreak of COVID-19 worldwide [1,2]. As of April 7, 2020, there were 1,279,722 human COVID-19 cases and 72,614 deaths worldwide [3], prompting public health interventions that mitigate transmission of the pandemic such as wearing face masks, practicing social distancing, and following home confinement recommendations. As China is a unitary one-party socialist republic with strong governmental control, entire cities in the Wuhan Province were locked down and underwent aggressive measures that brought the epidemic under control [1,4]. However, little is known about public health interventions in democratic countries.

The democratic republic of South Korea, one of the geographical neighbors of China, had the second highest number of COVID-19 cases until February 2020 [5]. However, with a well-organized testing program, contact tracing, strict case isolation, and public cooperation that included wearing masks and washing hands, Korea has emerged as a model country with exemplary public health interventions [6]. As of April 11, 2020, COVID-19 cases have dropped sharply, and only 30 new infections have been reported in South Korea since. Further, there have been no new infections in the Daegu Region, which had the highest proportion of COVID-19 cases (65% of South Korea's total number of cases) [3]. Therefore, epidemiological data and experience regarding the characteristics of SARS-CoV-2 transmission in Korea are valuable to find the right strategies to combat COVID-19.

Based on the experience with the Middle East respiratory syndrome (MERS) outbreak, South Korea has set up a novel monitoring system to collect information and manage patients with COVID-19 and their contacts by using GPD (cell phone location), card transaction logs, closed-circuit television (CCTV), and a history of medical facility use [7]. Using data acquired by this monitoring system, we investigated the epidemiological characteristics of SARS-CoV-2 transmission in South Korea and evaluated whether the social distancing campaign is effective in mitigating the spread of COVID-19.

## Methods

### Data Collection

Data were collected from individuals with laboratory-confirmed SARS-CoV-2 infection who subsequently completed the preliminary epidemiological surveillance conducted by each local government of South Korea (Seoul, Incheon, Sejong, Daegu, Gwangju, Ulsan, Busan, Gyeonggi-do, Gangwon-do, Chungcheongbuk-do [Chungbuk], Chungcheongnam-do [Chungnam], Gyeongsangbuk-do [Gyeongbuk], Gyeongsangnam-do [Gyeongnam], Jeollabuk-do [Jeonbuk], Jeollanam-do [Jeonnam], and Jeju) [8-12] and the Korea Centers for Disease Control and Prevention (KCDC) between January 19, 2020, and April 7, 2020. Epidemiological surveillance data were collected by epidemic intelligence service officers of each local government and the KCDC using the novel monitoring system that uses GPS (cell phone location), card transaction logs, CCTV, and a history medical facilities use. The study protocol was approved by the Institutional Review Board of Sejong University (SJU-HR-E-2020-003) and written informed consent was waived by the ethics commission, owing to the urgent need to collect data.

A cluster infection was defined as a group of similar COVID-19 cases that occurred in the same area during a short time interval. Nonclustered cases were patients with COVID-19 unrelated to any other patients with COVID-19 in time or place [13]. Laboratory confirmation of SARS-CoV-2 infection was defined as a positive result of real-time reverse transcriptase polymerase chain reaction assay of nasal or pharyngeal swabs, in agreement with the World Health Organization (WHO) guideline [14]. Information on age, sex, region of residence, and infection route was obtained for each participant. Death data as of April 7, 2020, were obtained by the KCDC.

### Statistical Analysis

We set January 19, 2020, as the index date (epidemiologic day 1) and April 7, 2020, as epidemiologic day 80. The primary endpoint was the mortality risk among participants with noncluster infection and those with cluster infection. Analysis of covariance was used to calculate the adjusted mean difference and 95% CI after adjustment. The following factors were considered potential confounders: age (0-19 years, 20-39 years, 40-59 years, and 60 years or older), sex, diagnosis date, and region of residence (urban [Seoul, Incheon, Sejong, Daegu, Gwangju, Ulsan, and Busan] vs rural [Gyeonggi-do, Gangwon-do, Chungbuk, Chungnam, Gyeongbuk, Gyeongnam, Jeonbuk, Jeonnam, and Jeju]).

Our secondary endpoint was whether a social distancing campaign was effective in mitigating the spread of COVID-19. We divided the population into two distinct periods: before the social distancing campaign (January 19, 2020, to March 22, 2020) and after the social distancing campaign (March 23, 2020, to April 7, 2020). We tested whether trends in newly confirmed COVID-19 cases changed after the social distancing campaign compared with those before the campaign. We implemented interrupted time series analysis to detect a change of slope after the launch of the nationwide social distancing campaign. We introduced the following equation to compare the effect of the campaign, where:


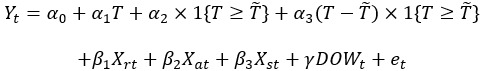


Y_t_ is the newly infected person on day t; T is the number of days elapsed from the first confirmed infectious case; 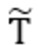 is the breakpoint day with the day when the nationwide social distancing campaign was launched (64); α_0_ is the number of infected patients on the first day of the infection; α_1_ is the slope of novel cases per day before the campaign; α_2_ is the newly infected cases at the start of the campaign compared to α_0_; α_3_ is the difference in novel infection rate before and after launching the campaign. Therefore, α_1_ + α_3_ is the trend of the number of daily new infections after the onset of the campaign. X_rt_, X_at_, and X_st_ are vectors each containing region specificity, age distribution, and gender composition of the patients on day t, and β_1_, β_2_, and β_3_ are the proportional coefficients of each covariate vector. DOW_t_ is the day of the week (eg, Saturday) on day t, *γ* is its coefficient, and e_t_ is an error term.

Network visualization was performed using Gephi version 0.9.2 [15]. The relative positions of nodes and edges were implemented by the Fruchterman-Reingold algorithm [16]. The algorithm would optimally draw the whole layout of the graph to cluster similar nodes and simplify the path of edges to express the transmission routes clearer. Next, we added the “Nooverlap” option to increase visibility further. The dot represented an individual and the line represented an individual tracing result. Larger dots represent clustered infections, where size was proportional to the number of infected individuals. Overseas influx and influx of community-acquired infections (Daegu and two cities in Gyeongbuk [Cheongdo and Gyeongsan]) connected 641 and 229 dots, respectively.

Each categorical value is reported as the number of patients (percentage). Statistical analyses were performed using SPSS version 25.0 (IBM Corp), and R software version 3.6.2 (R Foundation for Statistical Computing). A two-sided *P* value<.05 was considered statistically significant.

### Patient and Public Involvement

No patients were directly involved in designing the research question or conducting the research. No patients were asked to interpret or write up the results. There are no plans to involve patients or relevant patient communities in dissemination at this moment.

## Results

**Figure 1:**
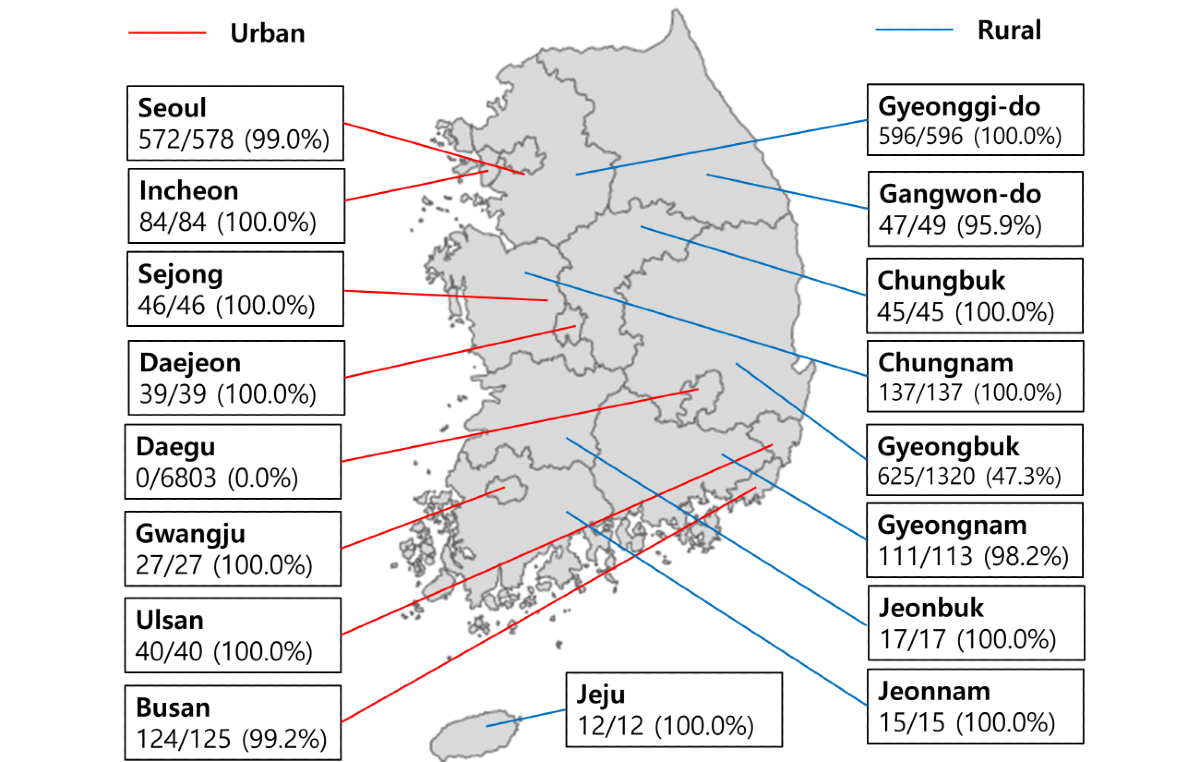
Our study population in each region (number of our study population/number of total patients with confirmed COVID-19). Of 9550 patients with confirmed COVID-19, there were 2134 patients with confirmed COVID-19 who completed the epidemiological surveillance.

From January 19, 2020, to April 7, 2020, there were 10,046 patients with laboratory-confirmed COVID-19 in South Korea. Among the 10,046 patients, 7509 were excluded for the following reasons: epidemiological investigation was not possible due to community-level outbreaks (Daegu and two cities in Gyeongbuk [Cheongdo and Gyeongsan]; n=7493) or because the epidemiological investigation was incomplete (n=16). The final sample size was 2537 (1160 men and 1377 women; Figure 1).

**Figure 2:**
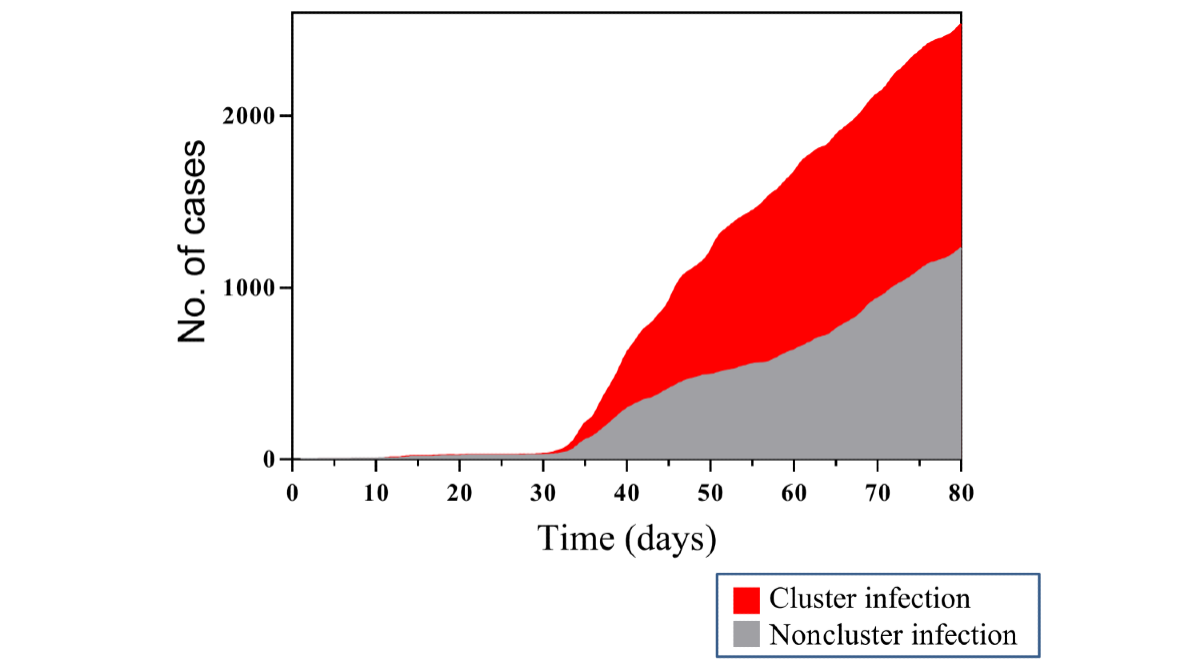
Number of infections based on infection type (cluster and contact cases vs noncluster cases) in South Korea from January 19, 2020, to April 7, 2020.

**Figure 3:**
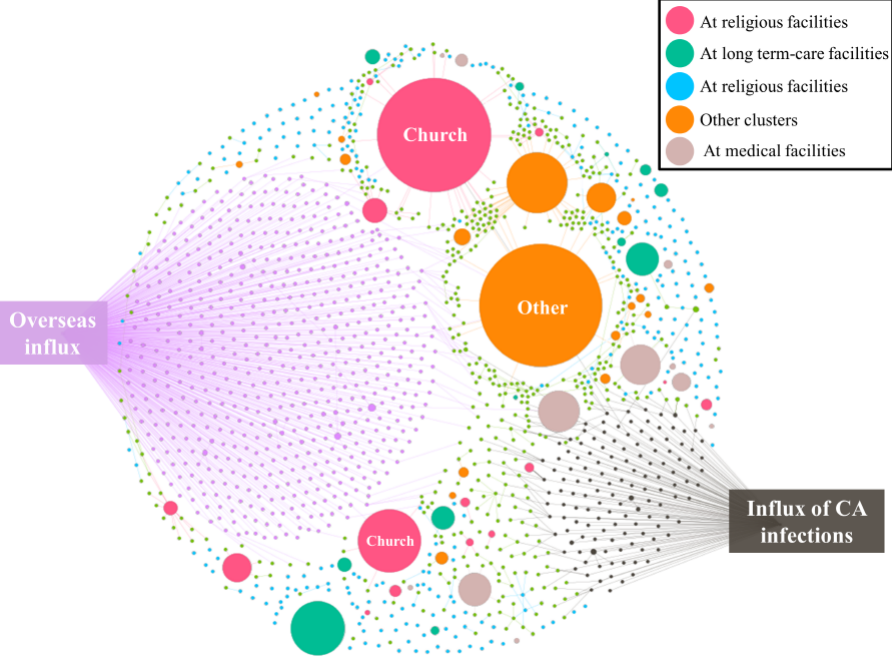
Infection spread network visualization of COVID-19 in South Korea from January 19, 2020, to April 7, 2020. Each dot represents an individual, and each line represents an individual’s tracing results. Overseas influx and influx of community-acquired infections (Daegu and two cities in Gyeongbuk [Cheongdo and Gyeongsan]) are shown by 641 and 229 connected dots, respectively. CA: community-acquired.

Table 1 shows the demographic characteristics of the participants. There were 1305 cluster cases (51.4%) and 1232 noncluster cases (48.6%; Figure 2). Cluster cases were linked to medical facilities (n=143, 5.6%), long-term care facilities (n=221, 8.7%), religious facilities (n=486, 19.2%), and other locations (n=455, 17.9%), which included military units, dance studios, karaoke bars, internet cafés, public transport, prisons, and the workplaces of each patient. Noncluster cases were linked to the overseas influx (n=641, 25.3%), influx in community-infection outbreak areas (n=229, 9.0%), and sporadic cases (n=362, 14.3%). Figure 3 and Multimedia Appendix 1 show the infection spread network visualization of COVID-19.

**Table 1:** Demographic characteristics of patients with confirmed COVID-19 in South Korea.

Characteristic		Entire cohort, n (%)	Cluster and contact cases, n (%)					Noncluster cases^a^, n (%)
			Linked to medical facilities	Linked to long-term care facilities	Linked to religious facilities	Others^b^		
Patients		2537 (100)	143 (5.6)	221 (8.7)	486 (19.2)	455 (17.9)	1232 (48.6)	
**Age (years)**								
	0-19	151 (6.0)	3 (2.1)	2 (0.9)	33 (6.8)	35 (7.7)	78 (6.3)	
	20-39	974 (38.4)	28 (19.6)	15 (6.8)	196 (40.3)	123 (27.0)	612 (49.7)	
	40-59	805 (31.7)	44 (30.8)	39 (17.6)	162 (33.3)	240 (52.7)	320 (26.0)	
	≥60	607 (23.9)	68 (47.6)	165 (74.7)	95 (19.5)	57 (12.5)	222 (18.0)	
**Sex**								
	Male	1160 (45.7)	49 (34.3)	68 (30.8)	228 (46.9)	176 (38.7)	639 (51.9)	
	Female	1377 (54.3)	94 (65.7)	153 (69.2)	258 (53.1)	279 (61.3)	593 (48.1)	
**Region of residence**								
	Urban	934 (36.8)	25 (17.5)	8 (3.6)	146 (30.0)	210 (46.2)	545 (44.2)	
	Rural	1603 (63.2)	118 (82.5)	213 (96.4)	340 (70.0)	245 (53.8)	687 (55.8)	
**Died**								
	No	2500 (98.5)	132 (92.3)	202 (91.4)	485 (99.8)	454 (99.8)	1227 (99.6)	
	Yes	37 (1.5)	11 (7.7)	19 (8.6)	1 (0.2)	1 (0.2)	5 (0.4)	

^a^Noncluster cases were linked to overseas influx (641/2537, 25.3%), influx for community-infection outbreak areas (229/2537, 9.0%), and sporadic cases (362/2537, 14.3%).

^b^Other facilities included military units, dance studios, karaoke, internet cafés, public transport, prisons, and workplaces of each patient.

Table 2 indicates the mortality rate of COVID-19 according to the infection route. The multivariable regression analysis showed that the mortality was significantly higher in cluster cases linked to medical facilities (11/143, 7.70% vs 5/1232, 0.41%; adjusted percentage difference 7.99%; 95% CI 5.83 to 10.14) and long-term care facilities (19/221, 8.60% vs 5/1232, 0.41%; adjusted percentage difference 7.56%; 95% CI 5.66 to 9.47) than in noncluster cases.

**Table 2:** Mortality rate for COVID-19 according to the infection route in South Korea (n=2134).^a^

Cases		Mortality percentage (95% CI)		Adjusted difference (95% CI)		*P* value	
Noncluster cases		0.41 (–0.25 to 1.06)		Reference			
**Cluster and their contact cases**							
	Linked to medical facilities		7.70 (5.78 to 9.61)		7.99 (5.83 to 10.14)		<.001
	Linked to long-term care facilities		8.60 (7.06 to 10.14)		7.56 (5.66 to 9.47)		<.001
	Linked to religious facilities		0.21 (–0.83 to 1.24)		–0.14 (–1.40 to 1.13)		.88
	Others		0.22 (–0.85 to 1.29)		–0.14 (–1.42 to 1.15)		.88

^a^Risk factors were adjusted by age (0-19 years, 20-39 years, 40-59 years, and 60 years or older), sex, diagnosis date, and region of residence (urban [Seoul, Incheon, Sejong, Daegu, Gwangju, Ulsan, and Busan] vs rural [Gyeonggi-do, Gangwon-do, Chungbuk, Chungnam, Gyeongbuk, Gyeongnam, Jeonbuk, Jeonnam, and Jeju]).

**Figure 4:**
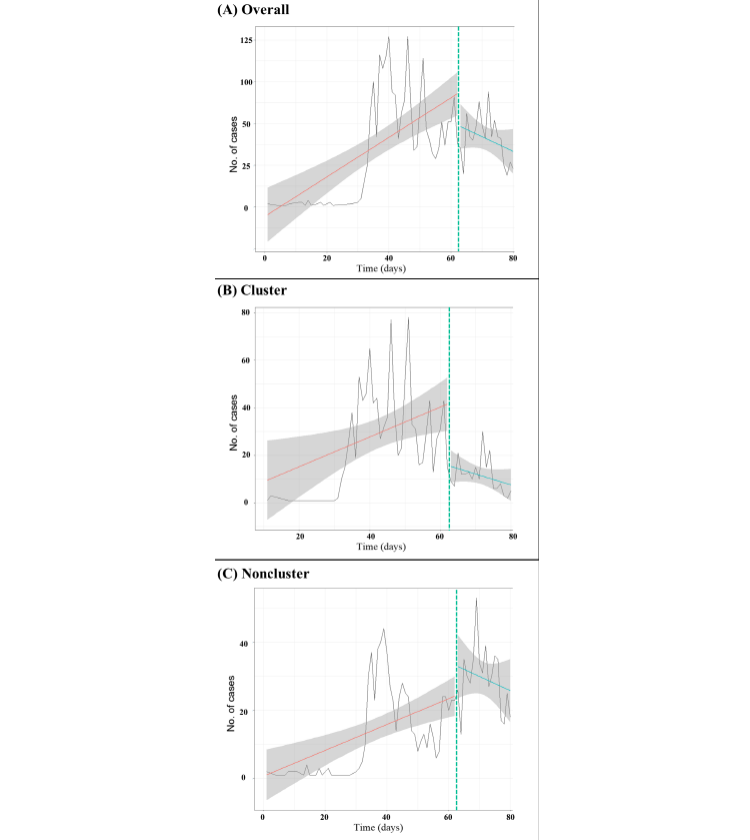
Number of new confirmed COVID-19 cases over the study period. The dashed vertical line at March 22, 2020, indicates the launch of the social distancing campaign. The solid red (before the social distancing campaign) and blue (after the social distancing campaign) lines represent the linear trends of new confirmed COVID-19 cases. Shaded areas represent 95% CIs for the linear trends.

Table 3 and Figure 4 show the trend in newly confirmed COVID-19 cases after the social distancing campaign by infection route. The trend was significantly negative in the overall population (adjusted trend difference –2.28; 95% CI –3.88 to –0.68) and the cluster infection group (adjusted trend difference, –0.96; 95% CI –1.83 to –0.09).

**Table 3:** New confirmed COVID-19 cases trends before and after a social distancing campaign in South Korea.^a^

Groups	Trend before the social distancing campaign (95% CI)	Trend after the social distancing campaign (95% CI)	Trend difference (95% CI)	*P* value
Overall	1.11 (0.62 to 1.59)	–1.18 (–2.70 to 0.34)	–2.28 (–3.88 to –0.68)	.005
Cluster	0.43 (–0.10 to 0.96)	–0.53 (–1.22 to 0.17)	–0.96 (–1.83 to –0.09)	.03
Noncluster	0.35 (0.16 to 0.54)	–0.34 (–1.34 to 0.67)	–0.69 (–1.71 to 0.33)	.19

^a^Risk factors were adjusted by age, sex, and region of residence.

## Discussion

### Principal Findings

To our knowledge, this is the first study to investigate the results of nationwide contact tracing of patients with COVID-19 and examine whether a social distancing campaign is effective in mitigating the spread of COVID-19. Cases of cluster infection and their contacts, which accounted for 51.4% (1305/2537) of the cases in this study, were linked to medical facilities, long-term care facilities, religious facilities, and other locations (military units, dance studios, karaoke bars, internet cafés, public transport, prisons, and workplaces of each patient). Moreover, COVID-19 linked to medical and long-term care facilities significantly increased the risk of mortality compared to noncluster COVID-19. Our study also showed that the social distancing campaign decreased the spread of COVID-19 in South Korea and differentially affected cluster infections of SARS-CoV-2. Therefore, strategies for the prevention of cluster infection of SARS-CoV-2 should be personalized and comprehensive, and multidisciplinary strategies to prevent COVID-19 should be developed. In particular, special attention should be paid to prevent cluster infections of SARS-CoV-2, especially in medical and long-term care facilities.

The pandemic spread of COVID-19 is exponentially escalating [17,18]. Cases of COVID-19 grew by several thousand each day in China in late January and early February, and took 2-3 days to double from 1000 to 2000 outside of China [1,17,19]. The velocity of the SARS-CoV-2 spread is substantially higher than that of the coronaviruses causing severe acute respiratory syndrome (SARS) and MERS (48 days for the first 1000 people to be diagnosed with COVID-19 compared to 130 days for SARS and 903 days for MERS) [2,20]. Aside from the characteristics of the virus itself, we investigated the epidemiological aspects of SARS-CoV-2 transmission in South Korea using contact tracing of confirmed cases and analyzed factors that may accelerate infection and death. We found three significant factors. First, cluster cases accounted for the highest portion of SARS-CoV-2–positive cases; second, overseas influx was significantly involved; and third, the majority of cases were confined to a specific area (Daegu Region).

An in-depth analysis of clustered cases revealed that a higher proportion of confirmed COVID-19 cases were related to religious, long-term care, and medical facilities. Cases from medical and long-term care facilities had a high mortality rate (11/143, 7.70% and 19/221, 8.60%, respectively) due to a higher proportion of vulnerable people including older adults and patients who are chronically ill present among these cases. These facilities are typically crowded with people in enclosed rooms, which create favorable conditions for transmission of respiratory diseases [21,22]. South Korea has the highest number of nursing hospitals (long-term care hospitals: 27.35 per 1000 people aged≥65 years) and the longest average length of hospital stay (average 18.5 days) of all Organisation for Economic Co-operation and Development countries [23]. Therefore, more care with strict regulation and quarantine programs should be applied to these kinds of facilities to avoid massive clusters of infection.

The enforced social distancing campaign was introduced by the Korean government on March 22, 2020. Our data support the enforced social distancing campaign as a highly effective method for preventing clustered infections. Our analyses demonstrated a significant reduction in clustered SARS-CoV-2 infections (adjusted trend difference –0.96; 95% CI –1.83 to –0.09) after the launch of the nationwide campaign. Since SARS-CoV-2 is transmitted via respiratory droplets [24,25], the purpose of the campaign was to keep a minimum distance to avoid transmission while maintaining personal hygiene. A droplet will fall under gravity or evaporate within 2 meters of the infected individual; therefore, staying 2 meters, or approximately three steps, away from other individuals will theoretically prevent droplet-induced transmission [26]. In addition to keeping personal distance, enforced social distancing includes following basic guidelines at work, religious facilities, sports and entertainment facilities, and other high-risk facilities, such as refraining from going outdoors when experiencing respiratory symptoms; having online gatherings instead of personal meetings; keeping a distance and avoiding talking when you eat; using personal belongings instead of sharing items; and keeping hand sanitizer available at entrances of buildings, elevators, and stairways.

It is interesting to note that the overseas influx had a significant role in the spread of the virus in South Korea. Recently, many countries have imposed government-issued international travel restrictions [27]. Although restricting travel may be useful in the early stage of the outbreak, it may be less successful once the outbreak is widespread [28]. Therefore, banning visitors from China or other COVID-19 high-risk countries to reduce the risk of reintroduction of the virus might be effective in countries that are at the early stage of the COVID-19 outbreak. However, for countries with a high incidence of COVID-19, an alternative strategy must be applied to mitigate SARS-CoV-2 transmission.

### Policy Implications

As the nature of COVID-19 is subclinical in some individuals, isolating early detected confirmed cases before transmission can occur is difficult [29]. Therefore, substantial effort should be made to prevent the virus from spreading by developing effective public health policy. First, public health policy should advise against social gatherings such as mass conferences, sporting events, musical concerts, and religious meetings. Instead, working remotely, online conferences, and online religious services should be encouraged. Second, strict screening and quarantine should be applied to those entering or leaving a region. Routine screening for SARS-CoV-2 and self-isolation should be required of visitors from areas of high incidence of COVID-19. Third, individuals should be advised against travel to regions of high COVID-19 incidence. Surveys of medical or long-term care facility visitors should be routinely conducted to screen for a history of visits to areas of high COVID-19 incidence. In addition, testing for COVID-19 should be required for patients and residents as well as staff and visitors in medical and long-term care facilities to prevent the introduction of COVID-19 in those facilities.

### Strengths and Limitations

First, as previously mentioned, one of the strengths of our study is that novel individual contact tracing data acquired by the KCDC and each local government in South Korea was used. By tracing individual data, we could categorize the source and characteristics of the transmission. Additionally, most other countries have not performed epidemiological surveys that include contact tracing; South Korea is thus far the only country to conduct epidemiological surveys with contact tracing. Therefore, we were able to identify the spread dynamics of COVID-19. Second, our study has a clear time point when a nationwide social distancing campaign was launched. Therefore, we could compare the trends of transmission before and after the campaign and evaluate the effectiveness of the public health intervention. Nonetheless, our study has some limitations. First, our data did not contain clinical information because we could not link hospital data to the epidemiological survey expeditiously. Second, we are still developing epidemiological surveys that include information on socioeconomic status (personal occupation and income) and time to development of COVID-19–related symptoms; hence, we were unable to analyze the time to symptom onset or socioeconomic status. Third, although the WHO stated that contact tracing includes the process of identifying, assessing, and managing people who have been exposed to a disease to prevent onward transmission [30], we only had tracing from confirmed cases; tracing for exposure remains for future study. Finally, epidemiological surveillance was not possible in some regions due to community-level outbreaks (Daegu and two cities in Gyeongbuk [Cheongdo and Gyeongsan]). Therefore, data from those regions were excluded.

### Conclusion

In this study, we investigated the nationwide contact tracing results of patients with COVID-19 and whether the social distancing campaign was effective in mitigating the spread of COVID-19. COVID-19 linked to medical and long-term care facilities significantly increased the risk of mortality compared with noncluster COVID-19. Moreover, our study shows that the social distancing campaign decreased the spread of COVID-19 in South Korea and differentially affected cluster infections of SARS-CoV-2. Therefore, our data may support driving public health policies in other countries and help normalize and restore social activities while minimizing the risk of transmission. Further cooperative global epidemic studies and updates are warranted to drive the best policy to control the transmission of SARS-CoV-2.

## Appendix

Multimedia Appendix 1 The dynamic infection spread network video of the COVID-19 in South Korea. The bottom bar represents the elapsed day after the first infection has occurred. The video includes the first 80 days of spread. Each line represents the spread occurrence between persons, and each dot represents the infected individual. Larger dots represent clustered infections, with dynamically increasing size, which means the number of infections in that cluster as time proceeds.

## Abbreviations

CCTV: closed-circuit television
KCDC: Korea Centers for Disease Control and Prevention
MERS: Middle East respiratory syndrome
SARS: severe acute respiratory syndrome
WHO: World Health Organization
